# Predictors of cervical tumour size for outpatients with cervical cancer at the University of Gondar referral hospital: a retrospective study design

**DOI:** 10.1186/s40001-023-01296-z

**Published:** 2023-10-24

**Authors:** Chalachew Gashu, Aragaw Eshetie Aguade

**Affiliations:** 1 Department of Statistics, College of Natural & Computational Sciences, Oda Bultum University, Chiro, Ethiopia; 2 Department of Statistics, College of Natural & Computational Sciences, University of Gondar, Gondar, Ethiopia

**Keywords:** Tumour size, Linear mixed model, Cervical cancer, Longitudinal data analysis

## Abstract

**Background:**

Cervical cancer is one of the most serious threats to women's lives. Modelling the change in tumour size over time for outpatients with cervical cancer was the study's main goal.

**Methods:**

A hospital conducted a retrospective cohort study with outpatients who had cervical cancer. The information about the tumour size was taken from the patient's chart and all patient data records between May 20, 2017, and May 20, 2021. The data cover 322 cervical cancer outpatients' basic demographic and medical information. When analysing longitudinal data, the linear mixed effect model and the connection between tumour sizes in outpatients were taken into consideration. A linear mixed model, a random intercept model, and a slope model were used to fit the data.

**Result:**

A sample of 322 cervical cancer outpatients was examined, and 148 (or 46% of the outpatients) tested positive for HIV. The linear mixed model with a first-order autoregressive covariance structure revealed that a change in time of one month led to a 0.009 cm^2^ reduction in tumour size. For every kilogramme more in weight, the tumour size change in cervical cancer patients decreased considerably by 0.0098 cm^2^. The tumour size change in the cervical cancer patient who was HIV-positive was 0.4360 cm squared greater than that in the HIV-negative outpatients.

**Conclusion:**

As a consequence, there was a significant association between the longitudinal change in tumour size and the predictor variables visit time, therapy, patient weight, cancer stage, HIV, oral contraceptive use, history of abortion, and smoking status.

## Introduction

It is a malignant tumour that has spread to the cervix and uterus. Initially, there can be virtually no symptoms. The main causes of cervical pre-cancer and carcinoma are one or more "high-risk" (or oncogenic) human papillomaviruses. The HPV infection has a protracted latency period. According to studies, genital HPV infection may occur in more than 80% of sexually active women at some point in their lifetime [1]. The cervical cancer epidemic in Africa is serious and complicated, with aetiologic factors, viral and non-infectious risk factors, and more. The dual burden of non-communicable and communicable diseases [2], challenges in providing preventive health services [3–5], a lack of health-related human resources [6], restricted access to treatment options, and low cervical cancer awareness among the general public and healthcare professionals [6] are just a few of the causes of the cervical cancer epidemic in Africa. There will be 342,000 cervical cancer fatalities by the end of 2020, with low- and middle-income nations accounting for 90% of these deaths [7]. There are schemes in place in high-income nations that enable women to receive the treatment they require, as well as frequent screenings and the HPV vaccine for girls. Pre-cancerous lesions can be found through screening at an early stage, when they are still treatable. Cervical cancer is frequently only identified after it has advanced and symptoms begin to appear, since access to these prophylactic treatments is limited in low- and middle-income nations. Additionally, there may be a lack of access to cancer treatments (such as chemotherapy, radiation therapy, and surgery), which could make the condition worse [8]. The 120,000 new instances of cervical cancer that are reported each year account for 20% of all cervical cancer diagnoses worldwide. In Africa, women make up a large part of those without access to treatment for cervical cancer. The recommended surgical treatment for early cervical cancer is a radical hysterectomy, but due to a lack of experience, many clinics and countries do not perform this procedure. Similar circumstances can be seen in a number of other African countries, some of which have no radiation protection at all. However, because they offer a higher standard of care, see many patients, and do so often, some institutions are better suited for clinical trial participation than others. There are a number of challenges in the treatment of cervical cancer in sub-Saharan Africa, including the lack of access to high-quality care (such as surgery, chemotherapy, and various types of radiation therapy), HPV and HIV prevention and screening, and appropriate imaging tests [9]. In Ethiopia, cancer accounts for about 5.8% of total national mortality. Except for Addis Ababa, where population-based data are available, there are thought to be about 44,000 cancer-related deaths annually, and 60,960 new instances of cancer are projected to be identified [10]. Every year, for every 100,000 people, 23% of Ethiopian women will develop cervical cancer, according to estimations. Cervical cancer is diagnosed in Ethiopian adult women more frequently than breast cancer, according to a study [8]. Ethiopia recorded 6294 new cases and 4884 fatalities from cervical cancer in 2018, one of the highest rates in the world [11, 12]. Numerous Ethiopian women are at high risk of acquiring cervical cancer, according to the statistics above. The bulk of them were carried out in Ethiopia and utilised logistic regression to assess knowledge [13], screening techniques using logistic regression [14], and Cox proportional hazard regression analysis to assess factors that affect the survival time of cervical cancer patients after diagnosis [1]. However, the study did not specifically mention weight or the frequent measurement of tumour size change in outpatients with cervical cancer, which is one of the major determinants determining prognosis. Inference and parameter estimation are biassed as a result of the failure to account for data correlation. Longitudinal studies are useful for modelling tumour size change and finding related risk factors over time because they take into consideration the correlation of tumour size within a patient. Missing data were discovered during the tumour size examinations; these problems with missing values were rectified using statistical methods. This study's goals were to identify risk factors that influence tumour size in outpatients with breast cancer and to model tumour size changes over time for these individuals. The results of this study provide recommendations for people engaged in patient care, therapy, and support, as well as for those creating efficient methods for tracking cervical cancer in outpatient settings. The findings of this study may also be used to raise public awareness of the factors that lead to the deaths of cervical cancer outpatients. Additionally, it enables us to share the findings with the Ethiopian Ministry of Health in order to support policymakers in educating the public about the factors that increase the risk of cervical cancer-related death, which may be avoided and treated if it is detected early and given the proper care.

### Methods

#### Study area

The study was conducted using information from the University of Gondar referral hospital in the Amhara National Regional State of Ethiopia, 720 miles northwest of Addis Abeba [15].

#### Data source and data collection procedure

Records of 322 outpatients with cervical cancer who were enrolled at the University of Gondar Teaching and Specialised Hospital in the Amhara area of northwest Ethiopia make up the longitudinal data used in this study. The study's data source was secondary data. Information about the tumour size was taken from the outpatient's chart, which contains clinical and sociodemographic data on every outpatient with cervical cancer who is receiving follow-up care. A checklist created by the outpatient department's follow-up format was used as a data extraction tool to pull data from an outpatient card for this study. The hospital's records of the patient's chart with cervical cancer under follow-up were used to gather information on variables connected to tumour size and associated factors. The healthcare workers at a cervical cancer clinic gathered the data for responses and covariates after getting adequate training on the study's variables. For all outpatients with cervical cancer who are being followed up, the chart includes laboratory and clinical data, as well as sociodemographic information.

#### Study design and population

This study used a retrospective cohort study design as its methodology. Only a few outpatients with cervical cancer were encountered by the researcher. As a result, the researcher decided against using a sample method for this investigation. All outpatients with cervical cancer who began their treatment at the University of Gondar referral hospital between May 20, 2017, and May 20, 2021, and who visited the department clinic at least twice more for prescription refills were included in this study.

##### Inclusion criteria

The study included patients who started treatment for cervical cancer at the University of Gondar referral hospital between May 20, 2017, and May 20, 2021 and who had at least two follow-up visits to the department clinic for prescription refills.

##### Exclusion criteria

The study excluded patients who were having treatment for cervical cancer while attending a clinic for prescription refills, those who had not returned for at least two follow-up visits, and those who were outside of the study period.

#### Dependent variables in the data

Results of the longitudinal measurement: The tumour size is measured roughly every 6 months. It is a continuous variable because it is measured in centimetres square.

#### Independent variables in the data

Age, place of residence, level of education, co-occurring conditions, a patient's history of abortion, visit time, HIV status, smoking status, weight, usage of oral contraceptives, stage, and histological type were considered independent variables that could affect the tumour size of outpatients with cervical cancer.

#### Longitudinal data analysis

The two sources of variation for longitudinal data are within-subject variation, or the fluctuation in measurements within each subject, which permits the analysis of changes over time, and inter-subject variation, or the variation in data between different individuals [16, 17].

Because the same subject is measured more than once at different points in time [18] and because measurements are taken on people who share a characteristic or category, which results in a correlation, longitudinal data analysis is related to observations of people over time. It is also feasible to have continuous or discrete binary longitudinal data [18]. The continual, repeated measurements (tumour size) were the main focus of this study.

#### Explanatory data analysis

Exploratory data analysis can be used to learn as much as you can about the raw data. Before performing any formal model fitting, it is recommended to first plot individual curves to carefully determine the data. To understand the data's variability and choose which random effects to include in the linear mixed model, individual profile plots and variance structures are used. In order to comprehend the time function that can be utilised to model the data, the mean structure was used [19]. Descriptive statistics and profile plots of tumour size across the research period were used in this work to investigate the data, and the nature of the data was evaluated by looking at individual profiles and the average evolution. We employed smoothing techniques to highlight the typical response as a function of an explanatory variable without relying on specific parametric models because our data are uneven and do not have equal visit times.

#### Linear mixed model (LMM)

Longitudinal data analysis is the result of numerous observations of the same subject made across time. Longitudinal response data may develop when measurements are conducted on the same subject repeatedly and on related topics. The response variables are probably related in both scenarios. While the continuous model within-subject variations was used to examine changes over time, longitudinal modelling between specific subject variations was carried out to investigate differences among people [17]. The longitudinal measurement of tumour size is modelled in the linear mixed-effects model as follows: *y*_*i*_ = *X*_*i*_β + *Z*_*i*_*b*_*i*_ + ε_*i*_,

where the (*n*_*i*_ × 1) vector of repeated measurements of tumour size for *i*th outpatients with cervical cancer is *y*_*i*_ = (*y*_*i*1_, *y*_*i*2_, *y*_*i*3_, … … …, *y*_*ini*_)′. A known design matrix known as *X*_*i*_ is *a n*_*i*_ × *p* corresponds to fixed predictors connecting *β* *to y*_*i*_. *β* is the *p* × 1 vector of the unknown population coefficient for the fixed effect. *Z*_*i*_ is a *n*_*i*_ × *q* design matrix that depicts the random elements that link *b*_*i*_ and *y*_*i*_. *q* × 1 vector of unobservable individual random effects parameter, *b*_*i*_ = (*b*_*i*1_, *b*_*i*2_, *b*_*i*3_, … … …, *b*_*iq*_)′. *b*_*i*_ = (*b*_*i*1_, *b*_*i*2_, *b*_*i*3_, … … …, *b*_*iq*_)′ is a vector of unobservable individual random effects with *q* 1. The *n*_*i*_ × 1 vector of unidentified random error is given by *ε*_*i*_ = (*ε*_*i*1_, *ε*_*i*2_, *ε*_*i*3_,, *ε*_*ini*_)′ [20].

#### Covariance structure

Comparability between measurements made on the same subject and those made on different subjects appears to be more common. Correlation is produced when measurements are repeated. For the analysis to be reliable, the variability between longitudinal measures must be well modelled. Compound symmetry (CS), unstructured (UN), Toeplitz (TOEP), and first-order autoregressive (AR (1)) are the four covariance structures that are most frequently utilised. We used the AIC or BIC value with the least value among those covariance structures to compare covariance [20].

#### Random intercept model

The random intercepts paradigm permits different intercepts between groups. A basic random intercepts model example that is used in the model fitting comprised clearly identifiable sections in particular [18]. These are the random and fixed components, respectively. The former is made up of the explanatory variable's coefficient and intercept. Two random terms make up the explanatory variable, ${\epsilon }_{i}\sim N\left(0,{\sigma }^{2}\right)$ and ${b}_{i}\sim N\left(0,{\sigma }_{b}^{2}\right)$. For separate subjects, the random effects ${b}_{i}$ and the within-subject error ${\epsilon }_{i}$ are independent, and for the same subject, they are independent of one another, i.e. $\mathrm{Cov}\left({b}_{i};{b}_{j}\right)=0$ if $i\ne j$, $\mathrm{Cov}\left({\epsilon }_{i},{\epsilon }_{j}\right)=0$ if $i\ne j$, and $\mathrm{Cov}\left({b}_{i};{\epsilon }_{i}\right)=0.$

$$y_{ij} = \beta_0 + \beta_1 x_{ij} + b_{0i} + \epsilon_i$$

#### Random slope and intercept model

The slope and intercept in this model are both arbitrary. Think about the straightforward random intercept and slope models, ${y}_{ij}={\beta }_{0}+{\beta }_{1}{x}_{ij}+{b}_{0i}+{b}_{1i}{z}_{ij}+{\epsilon }_{i}.$

From model y_ij_ we have ${b}_{1i}$, which represents the random slope effect of the coefficient ${b}_{ij},j=$ $1,\dots .{n}_{i}$ denotes the $j\mathrm{th }$tumour size on $i\mathrm{th}$ outpatients with cervical cancer. The random effects model covariance structure is

$$\left( {\begin{array}{*{20}l} {\beta_0 } \hfill \\ {\beta_1 } \hfill \\ \end{array} } \right) \sim N\left( {0,D_i } \right){\text{ with }}D_i = \left[ {\begin{array}{*{20}c} {\sigma_{b0}^2 } & {\sigma_{b0b1} } \\ {\sigma_{b0b1} } & {\sigma_{b1}^2 } \\ \end{array} } \right],$$

where ${\sigma }_{b0b1}$ denotes the covariance between the slopes and intercepts.

#### Parameter estimation for LMM

In addition to the fixed effect, mixed models also call for the estimation of random effects and the covariance structure of the random error. Restrictive maximum likelihood (REML) and machine learning (ML) are the two methods for parameter estimation most frequently used in this study. To estimate the LMM effects' parameters, machine learning is applied. By maximising the joint probability (likelihood function) given the data values, the maximum likelihood estimator (MLE) can be constructed. The maximum likelihood model uses the regression coefficients as known but fixed variables for computing the variance components, but it ignores the degrees of freedom lost during the estimation of the fixed effects. This leads to biassed ML estimations of the random effect variance. RML determines the variance components after eliminating the fixed effects from the model, which make up for the degree of freedom lost in estimating the fixed effects and result in a less skewed estimation of random effect variance.

#### Model selection

In order to select the most frugal model that best fits the available data, it is required to compare numerous models using a variety of methodologies and procedures. As a result, contrasting different models is essential for drawing conclusions from statistics [21]. The three most popular methods for model comparison are Akaike's Information Criterion (AIC), Bayesian Information Criterion (BIC), and likelihood ratio test (LRT). A better model to match the data might be possible with low values of AIC and BIC [20].

#### Goodness-of-fit test

A failure of this assumption affects the parameter estimates and residual effect standard errors in a linear mixed-effects model, which makes the assumption that random effects and residuals are normally distributed and uncorrelated with the error term. To ascertain whether these effects are typical and to identify any effect categories that are outliers, residual plots can be visually examined. It will be helpful to take a closer look at the plot of the fitted values by any relevant covariate against the standardised residuals [22]. To determine the viability of the within-group error assumption of normality, the residuals by variable normal quantile plot was used.

#### Treatment for missing data

A common issue in many real-world data sets is missing values. In longitudinal research, various techniques are employed to impute missing data. The most popular imputation method used to address missing variables is multiple imputation [23]. Missing data can be caused by a variety of things, such as survey question unanswered, measurement errors, and study participant dropouts. As part of the multiple imputations, which represent a range of potential values, each missing item is replaced by two or more acceptable values. The key advantage of the procedure is that an imputable data set has already been generated.

#### Operational definitions

Early-stage cervical cancer patients are those in stages I and II, whereas late-stage cervical cancer patients are those in stages III and IV [1, 5].

## Results

SPSS version 20 and R version 4.1.3 would be used to analyse the data. Table 1 displays the findings of the predictor variables for cervical cancer patients. A sample of 322 outpatients had a literacy rate of 189 (58.7%). 125 (30.8%) of 322 outpatients in a sample resided in a rural location. 178 (55.4%) out of a sample of 322 outpatients were smokers. Regarding the treatments, 125 (38.8%) were chemotherapy procedures, and 118 (36.7%) were radiation procedures. Of the 131 outpatients with cervical cancer, 131 (40.7%) were in the early stages. 184 (57%) of the 322 outpatients in the sample were HIV-negative. 34.5% of the 322 cervical cancer outpatients had never used an oral contraceptive. 189 (58.7%) of the cervical cancer outpatients had never had an abortion. 153 (47.5%) of the 322 outpatients with cervical cancer did not have any comorbid conditions. Of the 322 cervical cancer outpatients, 174 (54%) had an adenocarcinoma histology. 238 (73.9%) of the outpatients with cervical cancer were nonsmokers. Table 2 displays the patient findings for age, weight, and tumour size as continuous baseline data. The starting weight for the outpatients was 50.58 kg on average, with a 7.002 kg standard deviation. The average age at baseline was 75.24 years, with a standard deviation of 8.114 years. With an average tumour size of 26.16 cm square, the standard variation of the tumour size was 4.58 cm square. The minimum entrance age for the clinic treating outpatients with cervical cancer is 18.

**Table 1:** Descriptive result of categorical variables of cervical cancer, UoGRH, 2017–2021

Factor	Category	Total (%)
Residence	Rural	125 (38.8%)
	Urban	197 (61.2%)
Educational level	Literate	189 (58.7%)
	Illiterate	133 (41.3%)
Comorbid disease	No	153 (47.5%)
	Yes	169 (52.5%)
History of abortion	No	189 (58.7%)
	Yes	133 (41.3%)
HIV	No	184 (57%)
	Yes	138 (43%)
Stage	Early	131 (40.7%)
	Late	191 (59.3%)
Oral contraceptive uses	No	111 (34.5%)
	Yes	211 (65.5%)
Histology type	Adenocarcinoma	174 (54%)
	Squamous cell	148 (46%)
Treatment	Radiotherapy	118 (36.7%)
	Chemotherapy	125 (38.8%)
	Surgery	79 (24.5%)
Smoking status	No	238 (73.9%)
	Yes	84 (26.1%)

**Table 2:** Baseline traits of a continuous variable of cervical cancer*,* UoGRH, 2017–2021

Variables	N	Minimum	Maximum	Mean	Stand. deviation
Age in year	322	18	68	75.24	8.114
Weight in kg	322	36	60	50.58	7.002
Tumour size in cm^2^	322	0	49	4.58	26.16

### Distribution of tumour size

**Fig. 1:**
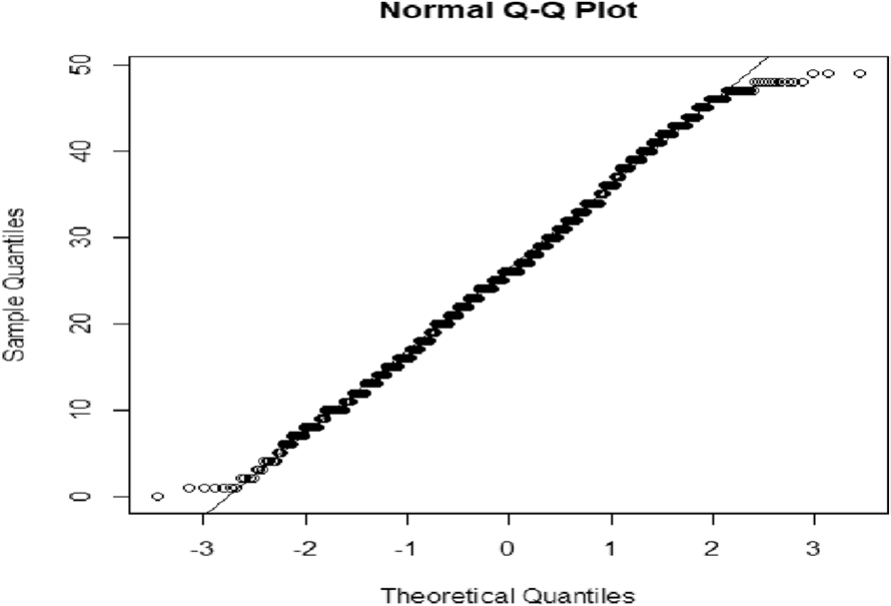
Q–Q plot of tumour size in cm.^2^

Figure 1 demonstrates that the points crossing the line in the tumour size data seem to support the assumption of normality.

### Exploratory (profile) data analysis

**Fig. 2:**
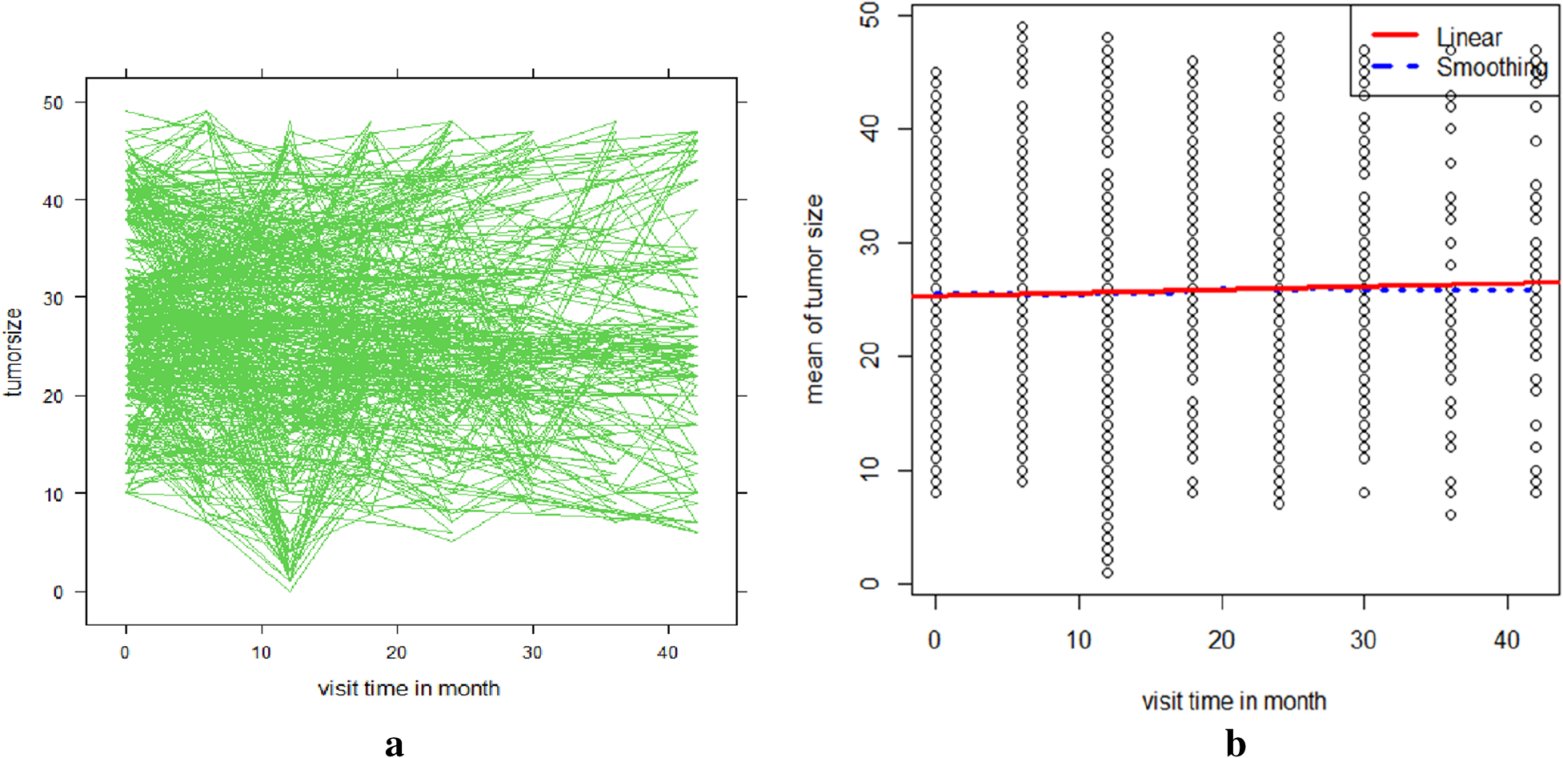
**a** Individual profiles with an average trend line, **b** loess smoothing plot with the average trend line of tumour size (cm.^2^)

Figure 2a shows that there was a change in tumour size over time and offers some information about outpatients with cervical cancer variability. It shows that patients had various initial tumour sizes and had various evolutionary processes over time. The random slope is also required in addition to the random intercept to allow subjects to have various slopes and account for the unstable between-subject heterogeneity at different time points. The mean structure of the tumour size is nearly linear over time, according to the smoothing plot in Fig. 2b, which was made using the Loess smoothing method. (That is, a linear link between visit duration and mean tumour size appears to exist.)

### Investigating the mean of tumour size structure for categorical factors

**Fig. 3:**
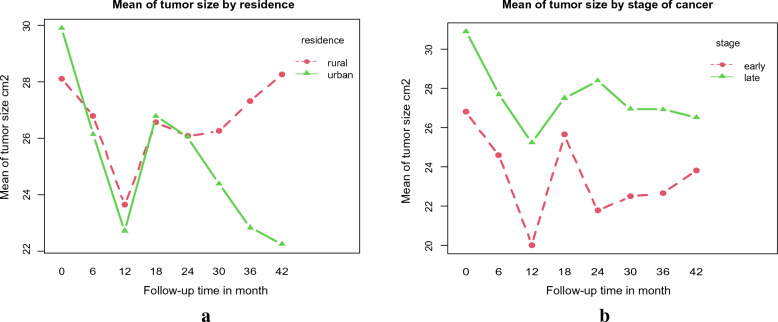
Mean profile plot of tumour size of outpatients with cervical cancer by **a**: place of residence, **b**: stage of cancer

The following plots were taken into consideration to examine the mean tumour size in relation to each categorical variable in the study over the course of visits: Fig. 3a shows that at baseline, cervical cancer patients in urban regions had larger mean tumour sizes than those in rural areas, but at the last follow-up (at 42 months), patients in rural areas had larger mean tumour sizes than those in urban areas. Figure 3b shows that mean tumour sizes were smaller in early-stage cervical cancer outpatients than in late-stage outpatients with cervical cancer.

### Selection of covariance structure in linear mixed model

Repeated measurements on the same patient have been found to be associated in many clinical trials; in this instance, the correlation and covariance among the repeated data should be precisely simulated. Fitting a mixed-effects model with each covariance matrix is the simplest technique to choose an acceptable covariance matrix structure. The best fit for the data is the one that has the minimum information requirements and corresponds to the mixed-effects model. As a result, linear mixed-effects models were fitted using the most commonly used covariance measures, and the model that produced the fit statistics with the least value was chosen.

The linear mixed-effects model that fits the autoregressive first-order covariance structure best, according to Table 3’s results, has the smallest BIC and AIC. This was due to the fact that when the distances between visits grew, the correlation between the repeated measurements of each participant diminished. So, using this framework, the subsequent analytical processes were carried out.

**Table 3:** Covariance structure for outpatients with cervical cancer, UoGRH, 2017–2021

Information criteria			
Covariate structure	AIC	BIC	Loglik
UN	3952.952	4040.375	− 1960.476
CS	3954.952	4047.839	− 1960.476
TOEP	3936.875	4028.673	− 1950.382
**AR (1)**	**3929.924**	**4022.811**	− 1947.962

Bold values indicate the best statistically significant results

### Choosing a random effect

To determine which random model to include in the model, we examined the AIC and BIC. Because the random intercept and slope model has a low BIC and AIC, it provides a better match for the data (see Table 4).

**Table 4:** Choosing the random effect to use in the LMM for outpatients with cervical cancer, UoGRH, 2017–2021

Information criteria			
Random effect	AIC	BIC	Loglik
Random intercept only	4082.298	4104.154	− 2037.149
Random slope only	4036.26	4074.507	− 2011.13
Random intercept and random slope**)**	**3916.88**	**4020.695**	− 1939.44

Bold values indicate the best statistically significant results

### Model selection

Table 5 shows that the full model was the fitted model that took into account all variables as opposed to the null model, which was the fitted model that did not take into account any covariates. (Small BIC and AIC values) The entire model produced a better fit to the data. A suitable model was chosen, and using the purposeful variable selection strategy, the variables from the univariate and fixed effects models were incorporated into the multivariable linear mixed effect model. The model was then fitted using the estimated values of the significant covariates.

**Table 5:** LMM comparisons for outpatients with cervical cancer, UoGRH, 2017–2021

Information criteria			
Model	AIC	BIC	Loglik
**Full**	**3916.88**	**4020.695**	− 1939.44
Null	4113.184	4135.04	− 2052.592

### Multivariable analysis of LMM

The corresponding *P*-value for the limited, restricted maximum likelihood estimates of variables and standard errors is shown in Table 6.

**Table 6:** Results of the final LMM for cervical cancer outpatients, UoGRH, 2017–2021

Covariate	Category	Estimate	Std. error	95% CI		*p*-value
				Lower	Upper	
	Intercept	4.56	0.25	4.07	5.05	**0.0001**
Treatment	(ref = radiotherapy)					
	Chemotherapy	0.17	0.08	0.008	0.33	**0.0401**
	Surgery	0.19	0.09	0.007	0.37	**0.0425**
Weight		− 0.009	0.004	− 0.02	− 0.0005	0.0396
Histology	(ref = Adenocarcinoma)					
	Squamous cell carcinoma	− 0.06	0.11	− 0.27	0.16	0.6104
Stage of cancer	(ref = early)					
	Late	0.22	0.09	0.04	0.40	**0.0198**
HIV	No					
	Yes	0.44	0.12	0.21	0.67	**0.0002**
Oral contraceptive use	(ref = No)					
	Yes	0.29	0.11	0.07	0.51	**0.0100**
Comorbid disease	(ref = No)					
	Yes	0.09	0.09	− 0.09	0.27	0.3321
History of abortion	(ref = No)					
	Yes	0.23	0.09	0.05	0.42	**0.0128**
Smoking status	(ref = No)					
	Yes	0.23	0.11	0.08	0.52	**0.0090**
visit time		− 0.009	0.002	− 0.012	− 0.006	**0.0001**
**Random effect**	**Std.Dev**	**95%CI**				
		**Lower**	**Upper**			
Intercept (*b*_0i_)	0.47	0.39	0.56			
Visit time (*b*_1i_)	0.01	0.001	0.02			
Corr (*b*_0i_, *b*_1i_)	0.58	0.30	0.93			
Residual (*ε*_*i*_	0.33	0.07	0.82			

Std.Dev = standard deviation. ref = reference category, bold indicates significance of variable at 5%

### Model diagnostics

**Fig. 4:**
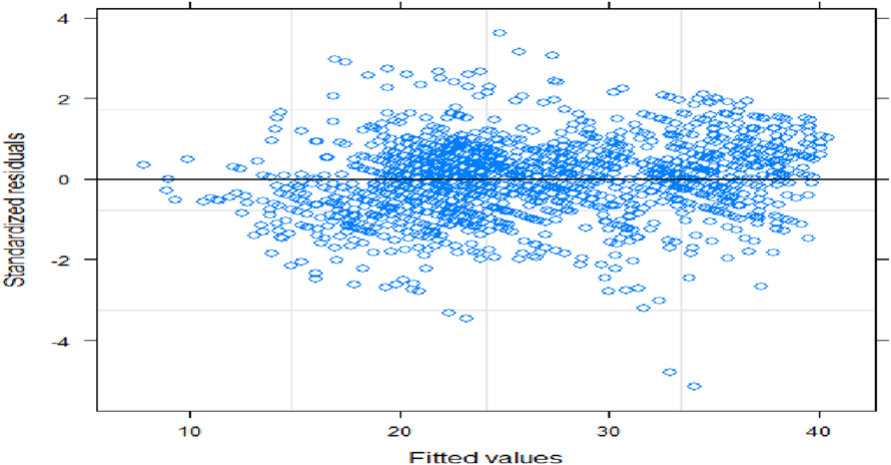
Tumour size residual plot for outpatients with data on cervical cancer*,* UoGRH, 2017–2021

Figure 4 illustrates how residual plots are used to evaluate the validity of model assumptions during model diagnostic checking for longitudinal data analysis. The "residual fit" concentrated around zero indicated that the linear mixed effect model was successful in fitting the tumour size data.

## Discussion

This study's major goal was to predict how cervical cancer outpatients' tumour sizes changed over time. The cervical cancer dataset was subjected to the linear mixed model, and the normality assumption held true. The data exhibited the best fit with the linear mixed-effects model matching the autoregressive first-order covariance structure. The data fit the random intercept and slope models better. Our results show that the slope and random intercept are connected, demonstrating the correlation between the slope and intercept of each individual. As a result, the variability within patients was 0.33, and the slope-to-intercept correlation was 0.582, indicating a favourable relationship between the intercept and slope of linear time. As a result, there was a 0.47 variability in intercept between individuals, and a 0.01 variability in slope. It is possible to assign 67% of the variation in tumour size that is not explained by the predictor variable to the subject.

The study found that women who took oral contraceptives were susceptible to cervical cancer and had no control over the size of the tumours. Condoms, pills, injectables, emergency contraception, implants, intrauterine contraceptives, the standard day method, the lactational amenorrhea method, and female and male sterilisation are all recommended as modern contraceptive methods to manage and control tumour size, whereas oral contraceptives increase tumour size, which is linked to cervical cancer and its complications. This result is consistent with [24]. As a result, there was a strong correlation between tumour size and disease stage in outpatients. The estimated tumour size change of the outpatient with cervical cancer in the late stage was considerably greater by 0.2226 cm square when compared to outpatients with cervical cancer in the early stage. It is advised that medical personnel pay closer attention to patients with advanced cervical cancer. This result is in line with work done in Ethiopia [25]. The results of the study showed that in outpatients with cervical cancer, the presence of HIV was a significant predictor of tumour size. In comparison to HIV-negative outpatients with cervical cancer, the estimated tumour size change of the HIV-positive patient was larger at 0.4360 cm square. This happens as a result of greater viral load counts and higher mortality rates in women with cervical cancer. This outcome is in line with [26]. The findings of this study showed that among outpatients with cervical cancer, a history of abortion was a significant prognostic factor for the advancement of tumour size. The estimated tumour size change was 0.2376 cm squared larger in cervical cancer patients with an abortion history than in patients without one. This outcome is consistent with [26]. According to the study's findings, the patients' survival duration was significantly predicted by comorbid disease. Cervical cancer outpatients who did not have comorbid disease fared better in terms of survival than those who did. This outcome is in line with [27]. In this study, visit time had a substantial detrimental impact on the evolution of tumour size. If the follow-up period was extended by one unit, there would be a 0.0084 reduction in the tumour size in outpatients with cervical cancer. This shows that a specific drop in tumour size occurred in those who had longer follow-ups. As the frequency of monthly follow-up visits declines, the condition gets worse. For every kilogramme more in weight, the estimated tumour size change in cervical cancer patients was considerably reduced by 0.0098 cm squared. The results show that weight is one of the key factors affecting the prognosis of outpatients with cervical cancer. The lack of pertinent predictor data is the study's main shortcoming. Since the data were taken from standard medical records, several variables could not be gathered because of technical difficulties. They were therefore not taken into account for this investigation. The quantity of sexual partners, the age of the first sexual encounter, and other factors fall under this category.

## Conclusion

Based on data gathered from the UOGRH for the period of May 20th, 2017 to May 20th, 2021, the study explores and identifies the parameters that are linked with repeated measurements of tumour size in outpatients with cervical cancer. This study reveals that a linear mixed effect model with a random intercept and random slope and a first-order autoregressive covariance structure is preferred for estimating the rate at which tumour size varies over the course of visits for outpatients. We found that the average tumour size pattern demonstrated a linear fall over time with respect to time, which was further corroborated by the model that displayed a negative estimate of time. This study found that 67% of the variation in tumour size was due to variations among outpatients. There was therefore a significant link between the predictor variables visit time, therapy, patient weight, cancer stage, HIV, use of oral contraceptives, history of abortion, smoking status, and the longitudinal change in tumour size. There was a high correlation between tumour size and the use of oral contraceptives, HIV status, cancer treatment, late cancer stage, and history of abortion, among other factors. At a 5% level of significance, however, there was an inverse relationship between baseline patient weight and visit duration and the size of cervical cancer tumours in outpatients.

### Limitations of the study

The use of secondary data, which could have added biases or inaccuracies, was one of the study's noted weaknesses. Another drawback was the absence of some variables from the medical records; future studies can take this into account to improve the analysis.

## Data Availability

The datasets utilised and analysed amid the current consideration are not freely accessible due to the information security of members, but are accessible from the corresponding author on sensible ask.

## Abbreviations

AIC: Akaike’s Information Criteria
BIC: Bayesian Information Criteria
CM: Centimetre
CIN: Cervical intraepithelial neoplasia
HIV: Human immunodeficiency virus
HPV: Human papillomavirus infection
ICO: Institut Català d’Oncologia
LMM: Linear mixed model
LRT: Likelihood ratio test
REML: Restricted maximum likelihood
ST: Score test
TASH: Tukir Anbsa specialized hospital
UoGRH: University of Gondar referral hospital
WHO: World Health Organization
